# The effect of performing mental exertion during cycling endurance exercise on fatigue indices: sex dependent differences

**DOI:** 10.3389/fphys.2025.1522626

**Published:** 2025-03-24

**Authors:** Hamid Amoozi, Hamidreza Barzegarpoor, Hamid Rajabi, Duane C. Button, Rana Fayazmilani

**Affiliations:** ^1^ School of Human Kinetics and Recreation, Memorial University of Newfoundland, St. John’s, NL, Canada; ^2^ Department of Biological Sciences in Sport, Faculty of Sports Sciences and Health, Shahid Beheshti University, Tehran, Iran; ^3^ Recovery and Performance Laboratory, Faculty of Medicine, L.A. Miller Center, Memorial University of Newfoundland, St. John’s, NL, Canada; ^4^ Sport sciences, Kharazmi University, Tehran, Iran; ^5^ Faculty of Medicine, Memorial University of Newfoundland, St. John’s, NL, Canada

**Keywords:** exhaustion, mental fatigue, rate of perceived exertion, women cyclists, stroop task

## Abstract

**Introduction:**

Men and women have different performance abilities, where women have greater tolerance for fatigue in long-distance exercise. Part of this greater tolerance may be due, in part, differences in men’s and women’s mental fatigue capacity during exercise. Thus, the aim of this study was to examine the effect of cycling endurance exercise, along with mental fatigue, on the sex dependent differences in exercise tolerance.

**Methods:**

Twenty-five (15 women and 10 men) cyclists in a randomized, controlled, and crossover protocol performed three experimental sessions. In the first session, the cycling peak power output (PPO) was determined and 65% of PPO was used for intensity of the experimental sessions. In sessions 2 and 3, participants performed Cycling + Mental Exertion (ME) (cycling endurance exercise with a ME-stroop task) and Cycling + Movie (cycling endurance exercise while watching a movie). Serum cortisol concentration and a psychomotor vigilance task (PVT) were measured pre‐ and post-sessions. During the experimental sessions rate of perceived exertion (RPE) and heart rate (HR) were recorded every 10 min and participants completed the NASA‐TLX questionnaire directly following the post- PVT.

**Results:**

Performing Cycling + ME was associated with a longer time to exhaustion (p < 0.05) and higher RPE following 40‐70 min in women than men (p < 0.05). Cycling + ME increased HR following 40-70 min in women (p < 0.05). For both men and women cortisol concentration level was higher pre‐ to post‐Cycling + ME with no difference between sexes. Women exhibited faster reaction times compared to men in both pre‐ and post‐sessions.

**Discussion:**

Performing mental exertion with cycling endurance exercise impairs endurance performance. While our findings align with some previous research, we suggest that men may be more prone to mental fatigue; however, further research needed to confirm this.

## Introduction

The ability to sustain aerobic exercise, also known as exercise capacity or exercise tolerance, is crucial for endurance athletes (Coyle et al., 1988; Glace et al., 2013). In long-duration exercise, the performance gap between men and women narrows, and women often have an advantage in long-distance events due to their greater resistance to fatigue (Glace et al., 2013; Froberg and Pedersen, 1984; Bam et al., 1997). Various physiological factors contribute to the difference in resistance to fatigue between sexes including blood flow, muscle perfusion (Hunter, 2014), skeletal muscle metabolism, fiber type properties (Hunter, 2014), and differences in regional brain activation (Bell et al., 2006; Wang et al., 2007). Some studies suggest that men and women experience a similar level of central fatigue in response to endurance exercise (Martin and Rattey, 2007; Russ et al., 2005). However, one possible reason for the disparity in fatigue levels could be psychobiological symptoms, such as mental fatigue (Yoon et al., 2009).

Mental fatigue is a psychobiological state that occurs during or after prolonged mental exertion. Mental exertion derived from different cognitive tasks is characterized by feelings of tiredness, lack of energy, and may result in decreased physical and cognitive performance (Ackerman, 2011). Performing mental exertion can lead to mental fatigue and activation of the pre-supplementary motor area and the anterior cingulate cortex (ACC) (Mostofsky and Simmonds, 2008). It has been found that rate of perceived exertion (RPE) is strongly linked with activity of this area (De Morree et al., 2012; Williamson et al., 2001; Williamson et al., 2002). As a result, an increase in RPE due to mental fatigue impairs endurance performance in activities such as running, cycling, and technical performance in soccer (Marcora et al., 2009; Smith et al., 2016; Pageaux et al., 2014). This suggests a psychobiological model of endurance exercise where higher RPE limits exercise tolerance (Marcora et al., 2008). Barzegarpoor et al. (2020) investigated the effect of simultaneous prolonged mental exertion and whole-body exercise on exercise performance in men cyclists. They found that mental fatigue decreased time to exhaustion by 31.8 min, along with higher RPE and stress hormones (prolactin and cortisol), compared with the control condition (Barzegarpoor et al., 2020). Furthermore, Lopes et al. (2020) indicated that performing an exhausting running endurance exercise subsequent to a 45-min Stroop’s colour-word conflict test (a cognitive task that measures selective attention, cognitive control, and processing speed (MacLeod, 1991) and is used to induce mental fatigue (Pageaux et al., 2014; Dallaway et al., 2022; Smith et al., 2019; Pageaux et al., 2014) induced a similar level of mental fatigue in women and men. During the time-to-exhaustion test, mental fatigue increased the RPE and also reduced time to exhaustion, similarly in women and men (Lopes et al., 2020). Yoon et al. (2009) investigated sex differences in response to mental fatigue (Mental-math task) during a fatiguing isometric contraction. Their study revealed that with performing a cognitive task, women had a shorter time to task failure and were more susceptible in fatigability for an isometric fatiguing submaximal contraction at 20% of maximum voluntary contraction (MVC) of the elbow flexors, which was due to increased sympathetic neural activity indices as well as cardiac work (Yoon et al., 2009). Furthermore, when women perform cognitive tasks, they have higher brain activation [especially in frontal activation and ventral anterior cingulate cortex (vACC)] (Butler et al., 2005). Higher brain activation is associated with a decrease in metabolic ratio (i.e., O_2_/glucose) (Fox and Raichle, 1986) which may increase glycolysis and fat metabolism. Thus, the mechanisms of fatigue and the higher perception of effort during endurance exercise are different between women and men. Hence, the combination of two different stresses (physical and mental) might cause an increase in the perception of effort, different brain area activation, and metabolism to limit endurance performance.

Despite the inconsistent findings that directly assessed mental fatigue simultaneously with endurance exercise, the primary aim of this study was to investigate the impact of simultaneous mental exertion during endurance cycling on exercise tolerance in both men and women, thereby enhancing our understanding of sex-specific fatigue responses. It was hypothesized that performing simultaneous cycling endurance exercise and mental exertion will impair time to exhaustion in men more than in women cyclists. A portion of the results on the male cyclist group in this study have been published previously (Barzegarpoor et al., 2020).

## Materials and methods

### Participants

Twenty-five endurance-trained cyclists (15 women: age = 23 ± 2 years; height = 166 ± 6 cm; weight = 60 ± 5 kg; Wmax = 257 ± 36 W and 10 men: age = 21 ± 3 years, height = 177 ± 6 cm, weight = 66 ± 8 kg; Wmax = 320 ± 31 W) were included in this study. Participants regularly participated in cycling endurance exercise (they cycled an average of about 150 km each week) and were apparently healthy with no history of chronic diseases or illnesses. Women participated in the study at various phases of their menstrual cycle. Nine women were in the follicular phase (up to 14 days after the onset of menstruation), and six women were in the luteal phase (between 14 and 28 days after the onset of menstruation). According to the guidelines of the university’s institutional ethical review board, each participant was informed both verbally and in writing about the risks of the research and gave informed consent to participate in the study. The study was approved by the Ethics Committee at Shahid Beheshti University, Iran (IR.SBU.ICBS 97/1033).

### Maximal cycling exercise protocol

In the first session, participants performed an incremental cycling test to determine their peak power output (PPO), which was defined as the maximum wattage (Wmax). The cycling exercise protocol was conducted on a cycle ergometer (Monark Ergomedic 839- Sweden) and started at 80 watts (W) for 3 min, and then the resistance was increased by 40 W every 3 min thereafter until exhaustion (defined as a cadence of less than 60 revolutions per minute (RPM) for more than 5 s despite strong verbal encouragement). The Wmax was calculated with the formula: Wmax = Wout + (t/180) × 40 [Wout: workload of the last completed stage; t: time (seconds) in the final stage] (Van Cutsem et al., 2017). The cycling ergometer was set in hyperbolic mode (i.e., the workload can be adjusted in watts) to allow participants to select their pedal frequency between 60 and 120 RPM. Saddle height was adjusted for each participant before the test and recorded for experimental sessions 2 and 3.

### Cognitive tasks

The mental exertion (ME) task was induced experimentally using a prolonged modified version of the Stroop colour-word task, which requires response inhibition and sustained attention. Briefly, the stroop task, which requires response inhibition and sustained attention, involved presenting colored words (red, blue, green, and yellow) on a computer screen where the buttons were located on the handlebar. In this task, each word was printed in a different ink color, with options including yellow, blue, green, and red (incongruent word-color combinations). The participant’s objective was to identify and indicate the ink color of the word presented, rather than considering the meaning of the word itself. However, if the ink color was red, the button to be pressed was the actual meaning of the word. The psychomotor vigilance task (PVT) was used to evaluate the effect of mental fatigue on the cognitive performance. The task was as follows: a red dot was shown in the middle of the computer screen for 500 ms at random intervals of 2–10 s for a total PVT duration of 5 min (50 stimuli). Participants were asked to use their dominant hand’s index finger to press a button as quickly and accurately as possible in response to the presented stimulus. To evaluate this test, reaction time (less than 500 ms) and the number of lapses were measured as key performance indicators. For more details on the mental exertion and PVT used in the current study see Barzegarpoor et al., (2020). The control session (i.e., no ME) included watching “When We Left Earth: The NASA Missions–Episode 6: A Home in Space” (Discovery Channel, United States) on the same computer screen.

### Heart rate (HR) and rate of perceived exertion (RPE)

Heart rate was continuously monitored throughout the experimental sessions using a HR monitor from Polar Electro OY Finland. Moreover, rate of perceived exertion was assessed using the Borg Rating of Perceived Exertion scale (Borg, 1998). This scale ranges from 6 (no exertion at all) to 20 (maximal exertion).

### National aeronautics and space administration task load index

The study used the National Aeronautics and Space Administration Task Load Index (NASA-TLX) rating scale to assess subjective workload. For more details on the NASA-TLX Questionnaire, see Barzegarpoor et al., 2020 (Psychological questionnaire) (Barzegarpoor et al., 2020). Briefly, after the Experimental protocols, participants completed the NASA-TLX which consists of six subscales measuring “mental demand”, “physical demand”, “temporal demand”, “performance”, “effort”, and “frustration”. The participants rated each item on a scale divided into 20 equal intervals anchored by a bipolar descriptor (e.g., high/low).

### Blood cortisol

When participants arrived at the laboratory, they were seated on a chair for 15 min and then the first blood sample was collected from the median cubital vein. The second blood sample was collected 30 min post cycling, coinciding with the peak concentration (Daly et al., 2005) in both the Cycling + ME and Cycling + Movie sessions. Then, plasma was isolated from the blood sample and stored at − 20°C for further analyzes. A human cortisol ELISA kit from ZellBio (ZB-11003-H9648) was used to determine plasma cortisol concentration.

### Study design

**FIGURE 1:**
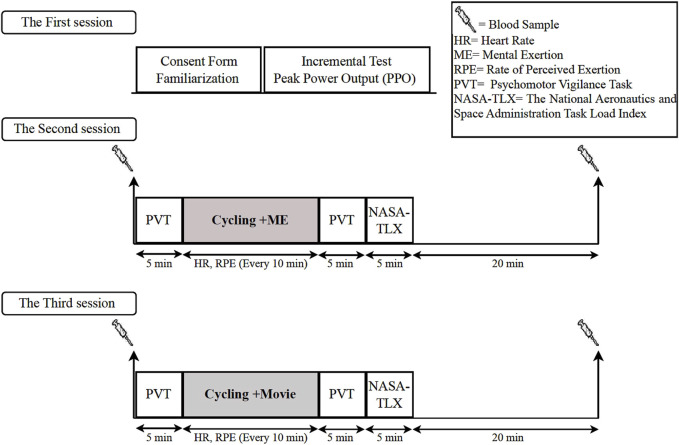
Overview of experimental protocol.

Participants completed three experimental sessions at the same time of day with at least 72-hours of rest between sessions. In the first session, all participants were informed about psychological measurements, then they completed the consent form and general health questionnaire, and their training history was recorded. Following this, the participants’ anthropometric index (height and weight) was recorded, and then they performed an incremental test to determine maximal wattage (Wmax). Participants then randomly performed sessions 2 and 3. These sessions were Cycling + ME (cycling endurance exercise simultaneously with the mental exertion-stroop task) and Cycling + Movie (cycling endurance exercise while watching a movie). Upon arrival at the laboratory for sessions 2 and 3, the first blood sample was collected. Following the blood collection, participants performed the PVT. After the completion of the PVT, participants performed a warm-up on a bike for 5 min at 60 W. Participants then completed either the Cycling + ME or Cycling + Movie protocol. The modified Stroop color-word task or watching a movie (the following section) was displayed on a monitor in front of participants while they were cycling. Cycling continued until exhaustion. Exhaustion was defined as the time point the participant could no longer maintain a cadence of 60 RPM that was sustained for more than 30 s, self-reported exhaustion, or a score of 18–20 on RPE. Following the cycling protocols, the post PVT was assessed. Directly following the PVT, participants completed the NASA-TLX questionnaire for 5 min and then the second blood cortisol samples were collected at 30 min post-cycling. Every 10 minutes throughout the experimental sessions, heart rate and RPE were recorded (Figure 1). Participants were instructed to follow guidelines that included avoiding intense exercise, alcohol, milk, caffeine, nicotine, and dairy products for 24 h before arriving at the laboratory to ensure adequate sleep.

### Statistical analysis

SPSS version 23 was used for statistical analyses. With a significance criterion of α = 0.05 and power = 0.80, the minimum sample size needed for this effect size was n = 24 for repeated-measures ANOVAs between-subject factors (Barzegarpoor et al., 2020; Jaydari and Lavender, 2019). The Shapiro-Wilk normality test was used to determine assumptions of normality. A Mauchly’s test was used to assess the assumption of sphericity. If this assumption was violated, the Greenhouse–Geisser correction was applied (Field, 2024). A three-way repeated-measures ANOVA was used to evaluate the effects of session (Cycling + ME vs Cycling + Movie), sex (men vs. women) and time (pre vs. post) on cortisol and PVT while HR and RPE evaluated at 10, 20, 40, 50, 60, and 70 min. A two-way ANOVA was used to evaluate the effects of session (Cycling + ME vs. Cycling + Movie) and sex (men vs. women) on time to exhaustion and NASA-TLX. A similar statistical analysis (i.e., three-way repeated-measures and two-way ANOVA) was applied to evaluate the effects of the menstrual cycle (luteal vs follicular) on all variables. When the ANOVA revealed a significant main and/or interaction effect, a Bonferroni *post hoc* test was used to identify differences. For all analyses, the level of statistical significance was set at *p* < 0.05. Data are represented by box and wisker plots or means ± standard deviations (SD) in figures. Partial eta-squared (pη2) measures indicating the magnitude of changes associated with significant main effects were provided and reported as small (<0.01), medium (≥0.06), or large (≥0.14).

## Results

### Time to exhaustion

**FIGURE 2:**
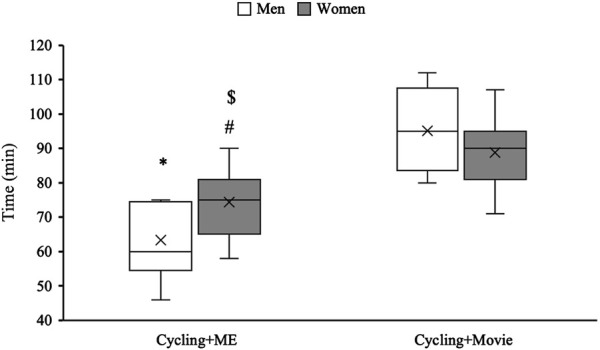
The effects of Cycling + ME and Cycling + Movie on time to exhaustion for Men (white bar) and Women (Grey bar). The asterisk (*) and hash (#) symbols indicate a significant within-subjects contrasts effect for session (p < 0.05). The dollar ($) symbol represents a significant (p < 0.05) difference between sex for Cycling + ME.

There was a significant reduction in time to exhaustion for men (F_1,22_ = 187.01, *p* < 0.01, ηp^2^ = 0.89) and women (F_1,22_ = 64, *p* < 0.01, ηp^2^ = 0.74) following the Cycling + ME. Specifically, time to exhaustion decreased 33% for men and 16% for women during Cycling + ME compared to Cycling + Movie (Figure 2). Furthermore, there was a significant difference in time to exhaustion between the sexes (F_1,22_ = 6.47, *p* = 0.018, ηp^2^ = 0.22), which time to exhaustion was 17% longer in Cycling + ME for women compared to men (74 ± 9 vs. 63 ± 11 min).

### Rate of perceived exertion

**FIGURE 3:**
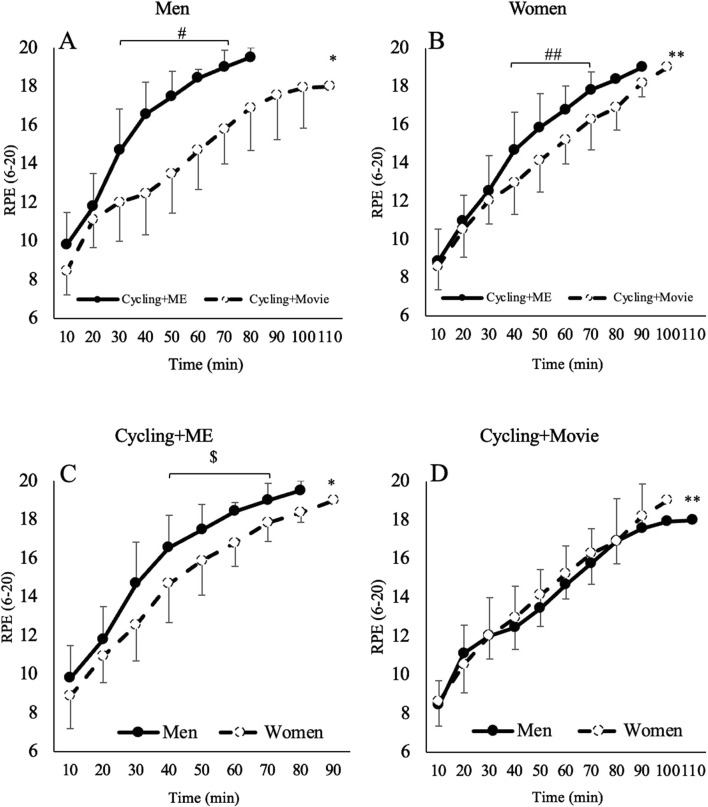
The effects of Cycling + ME (solid line) and Cycling + Movie (dashed line) on RPE for Men **(A)** and Women **(B)**. Regardless of session, RPE increased (*p* < 0.05) both men (represented by a single asterisk) and women (represented by an asterisk and double asterisk). The hash (#) and double hash (##) symbols indicate a significant within-subjects effect for session (*p* < 0.05). **(C, D)** indicate the effect of Cycling + ME and Cycling + Movie on RPE for men (solid line) vs*.* women (dashed line). The dollar ($) symbol represents a significant (*p* < 0.05) difference between sex from 40 to 70 min during the Cycling + ME session only.

RPE significantly increased for men following 30 (F_1,21_ = 20.54, *p* < 0.01, ηp^2^ = 0.49) and for both men and women following 40 (F_1,21_ = 46.56, *p* < 0.01, ηp^2^ = 0.68 and F_1,21_ = 14.78, *p* < 0.01, ηp^2^ = 0.41), 50 (F_1,21_ = 46.63, *p* < 0.01, ηp^2^ = 0.68 and F_1,21_ = 14.23, *p* = 0.01, ηp^2^ = 0.40), 60 (F_1,21_ = 49.45, *p* < 0.01, ηp^2^ = 0.70 and F_1,21_ = 12.78, *p* = 0.01, ηp^2^ = 0.37) and 70 (F_1,21_ = 37.87, *p* < 0.01, ηp^2^ = 0.64 and F_1,21_ = 17.52, *p* < 0.01, ηp^2^ = 0.45) minutes respectively, of Cycling + ME compared to Cycling + Movie. RPE was significant different between men and women following 40 (F_1,21_ = 5.98, *p* = 0.023, ηp^2^ = 0.22), 50 (F_1,21_ = 6.74, *p* = 0.017, ηp^2^ = 0.24), 60 (F_1,21_ = 18.35, *p* < 0.01, ηp^2^ = 0.46) and 70 (F_1,21_ = 13.52, *p* = 0.01, ηp^2^ = 0.39) minutes during Cycling + ME. Women’s RPE compared to men was 13%, 11%, 10% and 7% lower at 40, 50, 60, and 70 min, respectively during Cycling + ME (Figure 3).

### Heart rate

**FIGURE 4:**
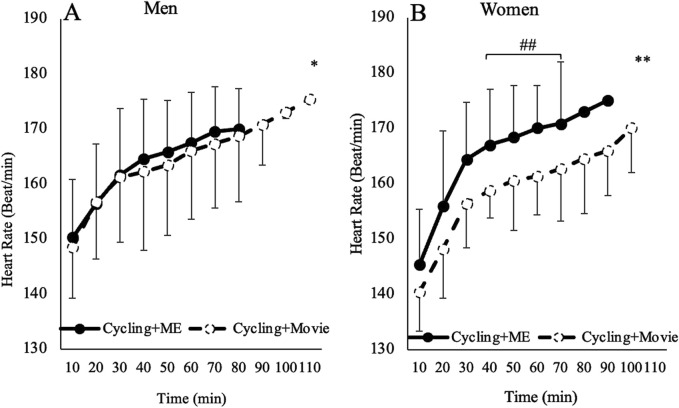
The effects of Cycling + ME (solid line) and Cycling + Movie (dashed line) on HR for Men **(A)** and Women **(B)**. Regardless of session, both men (represented by a single asterisk) and women (represented by a double asterisk) HR increased (*p* < 0.05). Double hash (##) symbols indicate a significant within-subjects effect for women in Cycling + ME compared to Cycling + Movie (*p* < 0.05).

Women had a significant increase in HR when performing Cycling + ME after 40 min compared to Cycling + Movie at 40 (F_1,14_ = 7.47, *p* = 0.016, ηp^2^ = 0.34), 50 (F_1,14_ = 5.84, *p* = 0.03, ηp^2^ = 0.29) 60 (F_1,14_ = 8.25, *p* = 0.012, ηp^2^ = 0.37) and 70 (F_1,14_ = 8.45, *p* = 0.011, ηp^2^ = 0.37) minutes (Figure 4).

### Cortisol

**FIGURE 5:**
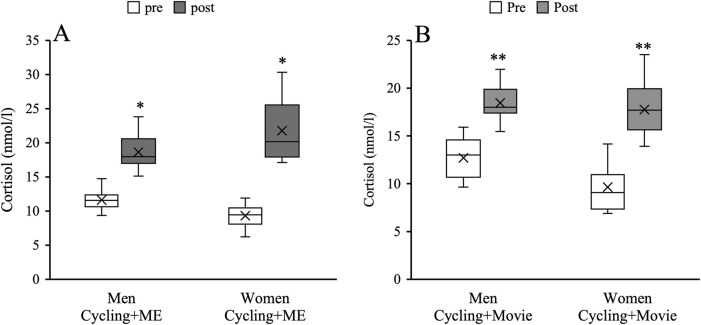
The effects of Cycling + ME **(A)** and Cycling + Movie **(B)** on cortisol concentration for men (white bar) and women (black bar). The asterisk (*) and the double asterisk (**) symbols indicate pre-post significant differences in Cycling + ME and Cycling + Movie for men and women, respectively (*p* < 0.05).

There was a significant main effect of time for Cycling + ME and Cycling + Movie on cortisol concentration in men (*Cycling + ME*: F_1,22_ = 15.75, *p* < 0.001, ηp^2^ = 0.41 and *Cycling + Movie*: F_1,22_ = 28.09, *p* < 0.001, ηp^2^ = 0.56) and women (*Cycling + ME*: F_1,22_ = 71.23, *p* < 0.001, ηp^2^ = 0.76 and *Cycling + Movie*: F_1,22_ = 137.19, *p* < 0.001, ηp^2^ = 0.86). Cortisol concentrations increased 37% and 57% for men and women, respectively (Figure 5). However, there were no sex differences on cortisol concentration (*p* > 0.05).

### National aeronautics and space administration task load index

There was a significant main effect of Cycling + ME on mental (F_1,22_ = 86.03, *p* < 0.001, ηp^2^ = 0.79) and physical demand (F_1,22_ = 18.54, *p* < 0.001, ηp^2^ = 0.45) for men and mental demand (F_1,22_ = 72.70, *p* < 0.001, ηp^2^ = 0.76) for women. Mental demand in Cycling + ME increased 135% for men and 41% for women compared to Cycling + Movie. However, there were no significant differences on any other NASA-TLX subscales between Cycling + ME and Cycling + Movie in both men and women (*p >* 0.05) (Table 1).

**TABLE 1:** The effects of Cycling + ME and Cycling + Movie on NASA-TLX (0–100) for men and women.

	Men			Women		
Workload (0–100)	Cycling + ME	Cycling + Movie	*p*	Cycling + ME	Cycling + Movie	*p*
Mental demand	87 ± 10	37 ± 15	<0.001*	89 ± 10	53 ± 18	<0.001**
Physical demand	61 ± 20	90 ± 6	<0.001*	77 ± 11	82 ± 16	>0.05
Temporal demand	65 ± 11	68 ± 21	>0.05	68 ± 21	72 ± 19	>0.05
Performance	75 ± 9	78 ± 7	>0.05	78 ± 7	83 ± 8	>0.05
Effort	82 ± 7	83 ± 19	>0.05	86 ± 7	79 ± 11	>0.05
Frustration	43 ± 17	36 ± 19	>0.05	30 ± 2	31 ± 9	>0.05

The asterisk (*) and double asterisk (**) indicates significant differences within-subject effect in mental and physical demand in Cycling + ME and Cycling + Movie for men and women, respectively (p < 0.05).

### The psychomotor vigilance task

A significant main effect on reaction time (RT) was observed for men from pre-to post-session for both Cycling + ME (F_1,23_ = 9.40, *p* = 0.005, ηp^2^ = 0.29) and Cycling + Movie (F_1,23_ = 16.76, *p* < 0.001, ηp^2^ = 0.42). However, in women, no significant change was observed in RT in both Cycling + ME and Cycling + Movie (*p* > 0.05). Moreover, a significant sex effect on reaction time was shown, indicating that women exhibited faster reaction times compared to men in both Cycling + ME (F_1,23_ = 63.01, *p* < 0.004, ηp^2^ = 0.73) and Cycling + Movie sessions (F_1,23_ = 51.90, *p* < 0.001, ηp^2^ = 0.69). The results also showed that women have a faster RT in pre-cycling compared to men in Cycling + ME (F_1,23_ = 7.23, *p* = 0.013, ηp^2^ = 0.23) and Cycling + Movie (F_1,23_ = 1038, *p* < 0.004, ηp^2^ = 0.31) (Table 2).

**TABLE 2:** The effects of Cycling + ME and Cycling + Movie on RT for men and women.

Group	Time	RT (ms)			Number of lapses		
		Men	Women	p	Men	Women	p
Cycling + ME	Pre	356 ± 47	312 ± 34#	0.01	1.3 ± 0.86	1.85 ± 0.96	<0.05
	Post	383 ± 27*	310 ± 2$	<0.001	4.1 ± 1.1*	1.23 ± 0.89$	<0.05
	*p*	<0.001	>0.05	**—**	<0.05	>0.05	**—**
Cycling + Movie	Pre	342 ± 42*	298 ± 31#	0.01	0.8 ± 0.78	1.01 ± 0.98	>0.05
	Post	377 ± 29	296 ± 18$	<0.001	3.3 ± 1.2*	1.45 ± 1.2$	<0.05
	*p*	<0.001	>0.05	**—**	<0.05	>0.05	**—**

The asterisk (*) indicates pre-post significant differences in Cycling + ME and Cycling + Movie for men (p < 0.05). The dollar ($) symbol represents a significant (p < 0.05) difference between sex. The hash (#) symbols indicate the significant difference between men and women in RT at baseline (p < 0.05).

### Menstrual cycle

There were no significant differences between the menstrual cycle phases (luteal and follicular) in Cycling + ME and Cycling + Movie for all independent variables (time to exhaustion: F_1,13_ = 1.03, p = 0.32, ηp^2^ = 0.074, RPE: F_6.72_ = 1.28, p = 0.29, ηp^2^ = 0.097, HR: F_6.54_ = 0.024, p = 0.96, ηp^2^ = 0.026, Cortisol: F_1,13_ = 0.19, p = 0.66, ηp^2^ = 0.015, NASA: F_6,65_ = 0.58, p = 0.71, ηp^2^ = 0.043, and PVT (RT): F_1,13_ = 0.008, p = 0.92, ηp^2^ = 0.001).

## Discussion

We aimed to investigate the impact of mental exertion during cycling endurance exercise on time to exhaustion and fatigue-related performance in endurance-trained cyclists and whether there were sex differences. The results showed that 1) time to exhaustion was decreased during cycling endurance exercise with mental exertion as opposed to watching a movie for both men and women but women were able to cycle longer before exhaustion occurred, 2) the longer time to exhaustion for women was associated with lower RPE compared men and 3) women had faster reaction time compared to men while performing simultaneous mental exertion and cycling endurance exercise. Thus, performing simultaneous mental exertion and cycling endurance exercise impairs time to exhaustion and overall performance in cyclists. While exercise impaired the performance in cyclists, women were impacted to a lesser extent than men in these exercise performance conditions. These findings emphasize the importance of considering sex differences in both mental and physical tasks when performing longer duration endurance exercise.

This study demonstrated that time to exhaustion was 17% longer in women compared to men while performing a mental exertion task along with cycling endurance exercise. These findings are in line with previous studies that showed time to exhaustion was reduced due to performing prolonged mental exertion before and during endurance exercise (Hunter, 2014; Marcora et al., 2009; Barzegarpoor et al., 2020). The difference in cycling duration for men and women may be due to physiological differences. The literature has shown that compared to men, women have a larger capacity for lipid metabolism, and a higher-density of type I fibers with widespread sympathetic-mediated actions on skeletal muscle, particularly β2 adrenergic receptors (Staron et al., 2000). Estrogen (17β-estradiol, E2) plays a significant role in enhancing lipid metabolism, which may influence substrate utilization during endurance exercise (Tarnopolsky, 2008). Although we found no significant differences in the menstrual cycles, the previous studies indicated that RPE varies across the menstrual cycle, with higher RPE observed during the early follicular phase (days 1–5) compared to the mid-luteal phase (Mattu et al., 2020; Xa et al., 2012), which may impact endurance exercise performance. Although these variables were not directly measured in our study, the aforementioned mechanisms may partially explain the observed sex difference in time to exhaustion. Beyond physiological factors, the psychological influences on endurance performance can be explored through the neurophysiological basis of RPE (i.e., afferent feedback and corollary discharge model) (De Morree et al., 2012). According to the corollary discharge model, the sensory signal from the central command is necessary to maintain the same level of force as fatigue occurs, leading to significant increases in RPE (Morree et al., 2012; Marcora et al., 2008). ACC, posterior cingulate cortex (PCC) (Maddock et al., 2001; Vogt et al., 1992), and prefrontal cortex (PFC) (Helton et al., 2010; Lorist et al., 2005) are potential areas implicated in this process. In the current study, women had a longer time to exhaustion (74 vs. 63 min), later and lower increases in RPE (following 40 vs 30 min) and they had a lower subjective workload (mental workload in NASA-TLX) compared to men in cycling endurance exercise with mental exertion. Fontes et al. (2013) used functional magnetic resonance imaging (fMRI) to evaluate the specific brain regions which activated during cycling exercise. They showed that cycling with RPE>15 associated with higher activity of PCC (Fontes et al., 2015). In addition, Williamson et al. (2002) indicated that during cognitive response inhibition tasks (i.e., the Stroop task), the activation of the ACC is strongly activated, and this brain area is related to the perception of effort. Contrary to the current results, Lopes et al. (2020) showed that performing a 45-min cognitive task (Stroop’s color–word conflict test) to induce mental fatigue or watching a 45-min documentary (as control) before a time-to-exhaustion test on a treadmill led to a similar increase in RPE and the same reduction in time to exhaustion in both men and women (Lopes et al., 2020). The contradiction between the current findings and those of Lopes et al. (2020) may be due to differences in the experimental protocols used to induce mental fatigue. During the current study the mental fatigue task occurred simultaneously with the physical exertion task whereas in the Lopes et al. (2020) study the mental exertion task took place prior to the physical exertion task. Furthermore, the mental exertion task was of a longer duration in the current study. Thus, the duration and application of when the mental exertion task is completed likely has a different impact on a time to exhaustion test.

Although there was no statistical sex differences in HR for men between sessions, women had a higher HR following Cycling + ME and Cycling + Movie. Women have a higher index of sympathetic nerval activity such as HR (Yoon et al., 2009). Higher sympathetic responses could impact neuromuscular function by influencing the blood flow and contractile function of skeletal muscle (Thomas and Segal, 2004). As a result, motor unit discharge rate and contractility of the muscle (type I, in particular) increase (Hunter, 2009; Roatta et al., 2008) and this may be, in part, one explanation for different time to exhaustion in men and women. Pageaux et al. (2013), Pageaux and Lepers (2018) speculated that sustained mental exertion might cause adenosine accumulation in the ACC, which would make endurance exercise feel harder than it was. Another possible explanation for the differences in time to exhaustion and RPE between men and women is that mentally fatigued women, with higher activity of premotor and/or motor regions as well as higher cerebral blood flow (Esposito et al., 1996), may experience a lower accumulation of metabolites (i.e., adenosine) (Roelands et al., 2020). When the activity of the brain increases, the metabolic ratio of O_2_ to glucose decreases, indicating a shift toward anaerobic glycolysis to meet elevated energy demands. This metabolic process results in the production of lactate, which can function as an alternative energy substrate for the brain. Furthermore, lactate may stimulate peripheral tissues to enhance fatty acid metabolism, thereby contributing to overall energy balance (Magistretti and Allaman, 2018). Thus, women may have higher brain activation and cerebral blood flow as well as lower RPE leading to a longer time to exhaustion than men. Women may have a psychological advantage in resistance to fatigue as we observed in the present study. While we did not assess cerebral blood flow, the change in metabolic ratio of O_2_ to glucose, or lactate, further investigation using neuroimaging techniques would help us to understand the underlying neurobiological mechanisms of sex differences in fatigue resistance during endurance exercise while being mentally exerted.

According to several studies, physical and psychological stress lead to higher cortisol secretion levels (Wright et al., 2012; Van Cutsem et al., 2017). In response to stress (physical or mental), in the CNS, cortisol controls its own secretion via a negative feedback loop by binding to different area of the limbic system (i.e., hippocampus, amygdala, and PFC). Animal studies suggested that the PFC plays an inhibitory role in regulating the HPA axis and the subsequent stress response (Herman et al., 2003; Herman et al., 2005). It is interesting to note that differences in men’s and women’s brain activity patterns following psychological stress have been linked to the right PFC. Wang et al (2007) indicated that an increase in cortisol AUC was associated with an increase in cerebral blood flow (CBF) in the right PFC and in the dorsal ACC for men and women, respectively. In addition, they observed the PFC (especially, the orbitofrontal area) was suppressed during and “post-stress” in men, whereas it only occurred during the stress task for women. Our results are in line with these sex-specific responses where women may experience a more acute but transient physiological stress response (higher heart rate during the task and higher cortisol post-task), while men may have a more prolonged neural and physiological response as indicated by sustained right PFC activity and continued OFC suppression (Wang et al., 2007). Thus, compared to men, women seem to have a physiological advantage for withstanding longer periods of moderate to high intensity endurance exercise while being mentally exerted.

The present findings demonstrated that performing simultaneous mental exertion and cycling endurance exercise increased accuracy and improved reaction time in the PVT pre-post cycling in women compared to men. Chmura et al. (1997) hypothesized that the positive effects of CNS activation with increasing catecholamine concentration are correlated with an improvement in cognitive performance. They demonstrated that during 20 min of exercise, reaction time is faster immediately following when the blood adrenaline threshold was reached (Chmura et al., 1997). Their hypothesis could be supported by indirect evidence which is related to greater activation in indexes of the sympathetic nervous system (i.e., HR) (Kajantie and Phillips, 2006) and CBF (Polich and Kok, 1995) potentially resulting in an improvement in cognitive performance. In addition, Jaydari Fard and Lavender (2019) showed a higher error rate during the first blocks (17 min) of the mental fatigue task, but it was similar for the second and third blocks between women and men. These results indicate that women need fewer cognitive resources to achieve the same level of cognitive performance as men (Li et al., 2006; Larson et al., 2011). Since some studies have shown that there are no differences in mental fatigue between women and men (Lopes et al., 2020; Jaydari and Lavender, 2019), it is possible that women may be less affected by mental fatigue over time when performing mental exertion tasks.

### Methodological considerations

When interpreting the current results, it is important to consider several factors. We did not monitor the number of errors made during experimental protocols. The number of errors potentially indicates a period of increased cognitive resource allocation for both men and women as they adapt to the cognitive task. Furthermore, we did not directly assess the impact of long-duration endurance exercise on corticospinal excitability. As fatigue decreases the muscles’ ability to generate force, it can reduce the excitability of the corticospinal pathway in transmitting neural signals from higher brain centers (motor cortex and spinal motoneurons) to the locomotor muscles. A lower level of excitability in the motor cortex and/or spinal motoneuron requires a higher synaptic input (i.e., greater central motor drive), which may result in an increase in RPE and impair whole body endurance exercise. Therefore, it is important to understand the impact of long-duration endurance exercise and the resulting fatigue on the integrity of the corticospinal pathway.

Several methodological considerations warrant attention to enhance the integrity of future research designs. In the current study the time to exhaustion was different in each session for men and women. This variability in exercise duration can influence physiological and psychological responses to mental fatigue. Future studies should match the exercise duration, perhaps by setting a fixed time limit or workload for each session. This would allow for a more direct comparison of physiological and psychological responses at a similar level of fatigue. In addition, replicating our design with the inclusion of error rate measurement may provide more conclusive evidence regarding 1) confirming whether women require a longer initial period of heightened cognitive resource allocation compared to men and 2) assessing if error rates converge for both sexes as the task progresses, suggesting similar performance levels despite potential initial differences in resource allocation.

## Conclusion

This study showed that long-duration mental exertion (stroop task) along with cycling endurance exercise decreased time to exhaustion presumably via changes in psychophysiological characteristics such as increased RPE, subjective workload, and likely changes in activation of the sympathetic nervous system which was indirectly assessed by increasing heart rate. It is interesting to note that while some research found no sex difference in mental fatigue (Lopes et al., 2020; Wang et al., 2007), our results, especially RPE and mental demand, indicated that for mental fatigue, longer duration is required to limit endurance exercise performance for women than men. Although it seems that the results of our research are somewhat similar to previous studies, there is no obvious functional theory to explain the resistance to mental fatigue between men and women. More research is required to overcome the limitations of this study, such as measuring further physiological parameters (adrenaline, blood lactate, and glucose concentrations), applying transcranial magnetic stimulation to investigate nervous system changes along the corticospinal tract, and investigating the number of errors and reaction time during mental exertion.

## Data Availability

The original contributions presented in the study are included in the article/supplementary material, further inquiries can be directed to the corresponding authors.
